# Exercise During Pregnancy and Prenatal Depression: A Systematic Review and Meta-Analysis

**DOI:** 10.3389/fphys.2021.640024

**Published:** 2021-06-28

**Authors:** Miguel Sánchez-Polán, Evelia Franco, Cristina Silva-José, Javier Gil-Ares, Javier Pérez-Tejero, Rubén Barakat, Ignacio Refoyo

**Affiliations:** ^1^AFIPE Research Group, Universidad Politécnica de Madrid, Madrid, Spain; ^2^Department of Education, Research Methods and Evaluation, Comillas Pontifical University, Madrid, Spain; ^3^Department of Physical Activity, Sports and Leisure Social Sciences, Universidad Politécnica de Madrid, Madrid, Spain; ^4^Department of Health and Human Performance, Universidad Politécnica de Madrid, Madrid, Spain; ^5^Department of Sports, Universidad Politécnica de Madrid, Madrid, Spain

**Keywords:** exercise, pregnancy, prenatal depression, model, fetus

## Abstract

**Background:** Prenatal depression is associated with an increased risk of physical, physiological, cardiovascular, and psychological diseases for mothers and future newborns. Prenatal depression and depressive symptoms could have negative effects on the cognitive, emotional, social, and behavioral development of children.

**Objective:** This study aimed to examine the influence of exercise during pregnancy on the prevalence of prenatal depression and depressive symptoms in the scientific literature.

**Data Sources:** A search was carried out examining different online databases up to November 2020.

**Methods of Study Selection:** A systematic review with random effects meta-analysis was performed. Only randomized controlled trials published in English or Spanish with pregnant populations and interventions with exercise programs carried out during pregnancy were included. The scores obtained by the tools that measured the emotional state and depressive symptoms as well as the number and percentage of depressed women of the study groups were analyzed.

**Tabulation, Integration, and Results:** We analyzed 15 studies and found a negative association between moderate exercise during pregnancy and prenatal depression (ES = −0.36, 95% CI = −0.58, −13, *I*^2^ = 80.2%, P_heterogeneity_ = 0.001). In addition, the studies also showed that women who were inactive during pregnancy had a 16% higher probability of suffering prenatal depression [RR = 0.84 (95% IC = 0.74, 0.96) *I*^2^ = 61.9%, P_heterogeneity_ = 0.010].

**Conclusion:** Supervised exercise during pregnancy may be useful for the prevention and reduction of prenatal depression and depressive symptoms.

**Systematic Review Registration:** Registered in PROSPERO (Registration No. CRD42020164819).

## Introduction

Depression (major depressive disorder) is an important, common, and serious medical illness that negatively affects how a person feels, thinks, and acts. Depression causes different symptoms, such as feelings of sadness, loss of interest/energy, difficulty thinking/concentrating, and/or thoughts of death or suicide. All symptoms can vary from mild to severe (Bienvenu et al., 2013).

During the last 20 years, the World Health Organization (2004) has monitored diseases associated with depressive symptoms and the dangerous growth rate of the prevalence of these diseases within developed countries. Pregnant women are not exempt from depressive symptoms, and pregnancy is a particularly vulnerable time for depression to occur compared with other periods of a woman's life (Campagne, 2004).

Indeed, the physical and psychological changes that occur in pregnant women are the largest promoters of this type of emotional lability (Lederman et al., 1997). Symptoms that alter emotional balance and lead to sickness (in some women) usually appear at the beginning of pregnancy; these symptoms, together with the fear of not being able to cope with the baby, hormonal changes, transformation of the female body, and the possible presence of a history of psychological disorders, lead to a complex condition and may be responsible for the well-known emotional lability during pregnancy (Barakat, 2006; Austin et al., 2008).

It seems that the relationships between gestational alterations of a psychological and emotional nature (depression, changes in self-esteem, anxiety, stress, insecurity, etc.) and physiological parameters (longer deliveries and more instrumental labors, altered birth weights, etc.) have been scientifically confirmed (Field et al., 2006; Rahman et al., 2007; Grote et al., 2010). This negative association extends beyond the gestational period, causing postnatal complications for both mothers and offspring (Hammond and Crozier, 2007; Deave et al., 2008; Hay et al., 2010; Field, 2011). Undoubtedly, the intrauterine environment is decisive for the life of future humans.

Recent studies have estimated the prevalence of depression during pregnancy to be between 10 and 30% (Teixeira et al., 2009). It is closely associated with depression in the postpartum period, which has a prevalence of between 17 and 17.7% (Hahn-Holbrook et al., 2018; Shorey et al., 2018).

Drug treatment during pregnancy is difficult and often questioned due to the possible side effects of antidepressants in the mother and fetus (Hammond and Crozier, 2007). Depression is a difficult complication to control because it is necessary to implement an intervention that avoids the possible negative effects on the fetus and the mother, such as altered brain development (O'Connor et al., 2002; Lee et al., 2007), an increased risk of preterm birth or intrauterine growth restriction (Field et al., 2006; Li et al., 2009; Field, 2011). Notwithstanding the aforementioned difficulty, it is clear that it is necessary to establish strategies that prevent the already proven, dangerous, and increasing association between prenatal depression and postpartum depression, which is a more well-known type of depression that involves a series of related complications, such as mother–child bonding difficulties (Wisner et al., 2009), infant feeding difficulties and infant overweight problems (Ertel et al., 2010), low birth weight and long hospital stays.

This situation constitutes an interesting incentive to investigate alternative treatments for depression (Hammond and Crozier, 2007). According to a review of studies about the effects of exercise on the non-pregnant population, previous studies report beneficial antidepressant effects (Barbour et al., 2007; Blumenthal et al., 2007; Martinsen, 2008). Furthermore, the results of previous investigations, involving psychological variables during an exercise program carried out in pregnant women are encouraging (Koniak-Griffin, 1994; Goodwin et al., 2000; Nordhagen and Sundgot-Borgen, 2002; Orr et al., 2006).

Over the last 30 years, exercise has been shown to have many benefits for pregnant women without a risk of adverse effects for maternal–fetal well-being provided that the activity is of a moderate intensity and is supervised by a professional. Although the scientific literature is not fully conclusive, there are many investigations indicating the positive effects of moderate exercise on the prevention of complications, including the adequate control of certain maternal, fetal, and newborn parameters (Barakat et al., 2015, 2016; Klein et al., 2018). However, due to fear, ignorance, or other factors, the prevalence of women who achieve the minimum weekly recommendation of exercise during pregnancy is ~15–20% (Mottola et al., 2018; Barakat et al., 2019).

The purpose of this systematic review and meta-analysis was to synthesize the literature and determine the effect of exercise during pregnancy on the prevalence of prenatal depression.

## Methods

This review was performed in accordance with the Preferred Reporting Items for Systematic Reviews and Meta-Analyses (PRISMA) guidelines (Moher et al., 2015) and was registered with PROSPERO, the International Prospective Register of Systematics Reviews (Registration No. CRD42020164819).

### Eligibility Criteria

For this systematic review, the PICOS framework (population, intervention, comparison, outcome, study design) was used, and the nature of the interventions included in this research (Moher et al., 2015) was analyzed. Only articles written in English or Spanish and published between 2010 and 2020 were selected.

### Population

The chosen population was pregnant women without any contraindication during pregnancy to undergo an exercise intervention during this period. Women suffering any absolute (e.g., heart failure, multiple pregnancy, or premature labor) or relative (e.g., essential arterial hypertension, cardiac arrhythmia, or anemia) contraindication were excluded from analyses.

### Intervention

The following characteristics of the interventions were analyzed: (i) intensity: except for one study, all of the included studies had a light-to-moderate intensity of the load, which was prescribed using 55–65% of maximum maternal heart rate and in some cases by means of the perception of effort (range 12–14 of the Borg Scale); (ii) duration of the program; (iii) type of exercise (e.g., aerobic, strength, balance, or pelvic floor training); (iv) weekly frequency of the sessions; (v) duration of the sessions; (vi) whether the exercise program was supervised by a professional; (vii) adherence of the sample to the exercise program; and (viii) in some studies analyzed, a complementary intervention with different outcomes was carried out, and so this was classified as “exercise + cointervention” as shown in Table 1.

**Table 1:** Characteristics of the studies analyzed.

**Author, year, and country**	**N; IG; CG**	**Intervention Physical exercise program**							**Main variables analyzed**	**Secondary variables analyzed**	**Co-intervention**
		**W Freq**.	**Int**.	**Time**	**Type**	**Sup**.	**Duration**	**Adh**.			
Daley et al. (2018) (United Kingdom)	784; 391; 393	2	Mod	30 min	Aerobic	Yes	8 weeks	–	Prenatal and postnatal depression	Maternal parameters and smoking	Smoking cessation and mental health treatment
Vargas-Terrones et al. (2019) (Spain)	124; 70; 54	3	Mod	60 min	Aerobic + strength + balance + pelvic floor + stretching + relaxation	Yes	22–26 weeks	69.3%	Prenatal depression and maternal parameters	Habits before pregnancy	Basic prenatal care
Haakstad et al. (2016) (Norway)	105; 52; 53	2	Mod	60 min	Aerobic + strength + relaxation	Yes	12 weeks	80%	Prenatal depression and regular physical exercise	Sociodemographic variables, habits, and complications during pregnancy	No
Perales et al. (2016) (Spain)	241; 120; 121	3	Mod	55–60 min	Aerobic + strength + stretching + relaxation	Yes	~30 weeks	90 ± 8%	Maternal variables, hypertension, and excessive weight gain	Prenatal depression and diabetes	No
Uebelacker et al. (2016) (United States)	20; 12; 8	1	Mod	75 min	Yoga	Yes	9 weeks	80%	Quality of life and prenatal depression	Sociodemographic variables and maternal parameters	Yoga practice at home
Davis et al. (2015) (United States)	46; 23; 23	1	Mod	75 min	Yoga	Yes	8 weeks	–	Prenatal depression and anxiety	Maternal parameters	No
Perales et al. (2015) (Spain)	106; 52; 54	3	Low–Mod	55–60 min	Stretching + aerobic + relaxation	Yes	~30 weeks	85%	Prenatal depression and maternal parameters	Habits in pregnancy and sociodemographic variables	No
Ussher et al. (2015) (United Kingdom)	785; 392; 393	2	Mod	30 min	Aerobic	Yes	8 weeks	88.5%	Prenatal depression	Maternal parameters	No
Perales et al. (2014) (Spain)	167; 90; 77	3	Low–Mod	55–60 min	Aerobic + strength + stretching + relaxation	Yes	~30 weeks	85%	Prenatal depression	Sociodemographic and maternal variables and new-born outcomes	No
Field et al. (2013b) (United States)	92; 46; 46	1	Mod	20 min	Yoga	No	12 weeks	–	Prenatal, postnatal depression, and anxiety	Cortisol, estriol, and progesterone levels	No
Field et al. (2013a) (United States)	92; 46; 46	1	Mod	20 min	Tai chi/Yoga	Yes	12 weeks	–	Depression and anxiety during pregnancy	Psychotic disorders and sleep complications in pregnancy	No
Satyapriya et al. (2013) (India)	96; 51; 45	7	Mod	60 min	Yoga	Yes	16 weeks	-	Maternal parameters, prenatal depression, and anxiety	Sociodemographic variables	No
Field et al., 2012 (United States)	84; 28; 28-28	2	Mod	20 min	Yoga	Yes	12 weeks	80%	Depression and anxiety during pregnancy	Legs and back pain	No
Robledo-Colonia et al. (2012) (Colombia)	80; 40; 40	3	Mod–High	60 min	Aerobic + stretching + relaxation	Yes	12 weeks	–	Depressive symptoms	Sociodemographic variables	Physiotherapy treatment
Mosquera-Valderrama et al. (2012) (Colombia)	74; 37; 37	3	Mod	50 min	Aerobic + stretching	Yes	12 weeks	–	Depressive symptoms	Sociodemographic variables	Walks twice a week unsupervised

*Rf, reference; RCT, randomized controlled trial; IG, intervention group; CG, control group; W freq., weekly frequency; Int., intensity; Mod, moderate; Sup., supervised sessions; Adh., adherence*.

### Comparison

Women who engaged in exercise or physical activity were compared with those who did not. Additionally, the intervention characteristics were reviewed (shown in Table 1) to enrich our understanding of each study: no exercise intervention; weekly frequency, duration (both the program and sessions) or supervision of the program by a professional; mode of exercise; or if depression was a primary or secondary variable.

### Outcomes

Target outcomes were the number of pregnant women with depression in both groups (to compare both the control and intervention groups) and the prenatal depression score of each depression questionnaire administered. The questionnaires dealing with prenatal depression among the selected articles were, in order of relevance, the Center for Epidemiologic Studies Depression Scale (CES-D), Edinburgh Postnatal Depression Scale (EPDS), The Short Form 36 Health Survey (SF-36) and Hospital Anxiety and Depression Scale (HADS).

### Study Design, Information Sources, and Search Strategy

To perform this review, the SPORTDiscus, ClinicalTrials.gov, and MEDLINE (PubMed) databases were searched at the Universidad Politécnica de Madrid. The search began in October 2019 and the study was last updated between February and March 2020.

- English: exercise OR physical activity OR sport OR fitness AND pregnancy OR prenatal depression OR depression OR emotional OR emotional factors AND randomized clinical trial.
- Spanish: ejercicio O ejercicio físico O actividad física O deportes Y embarazo O depresión prenatal O depresión O emocional O factores emocionales Y ensayo clínico aleatorizado.

### Study Selection

**Figure 1:**
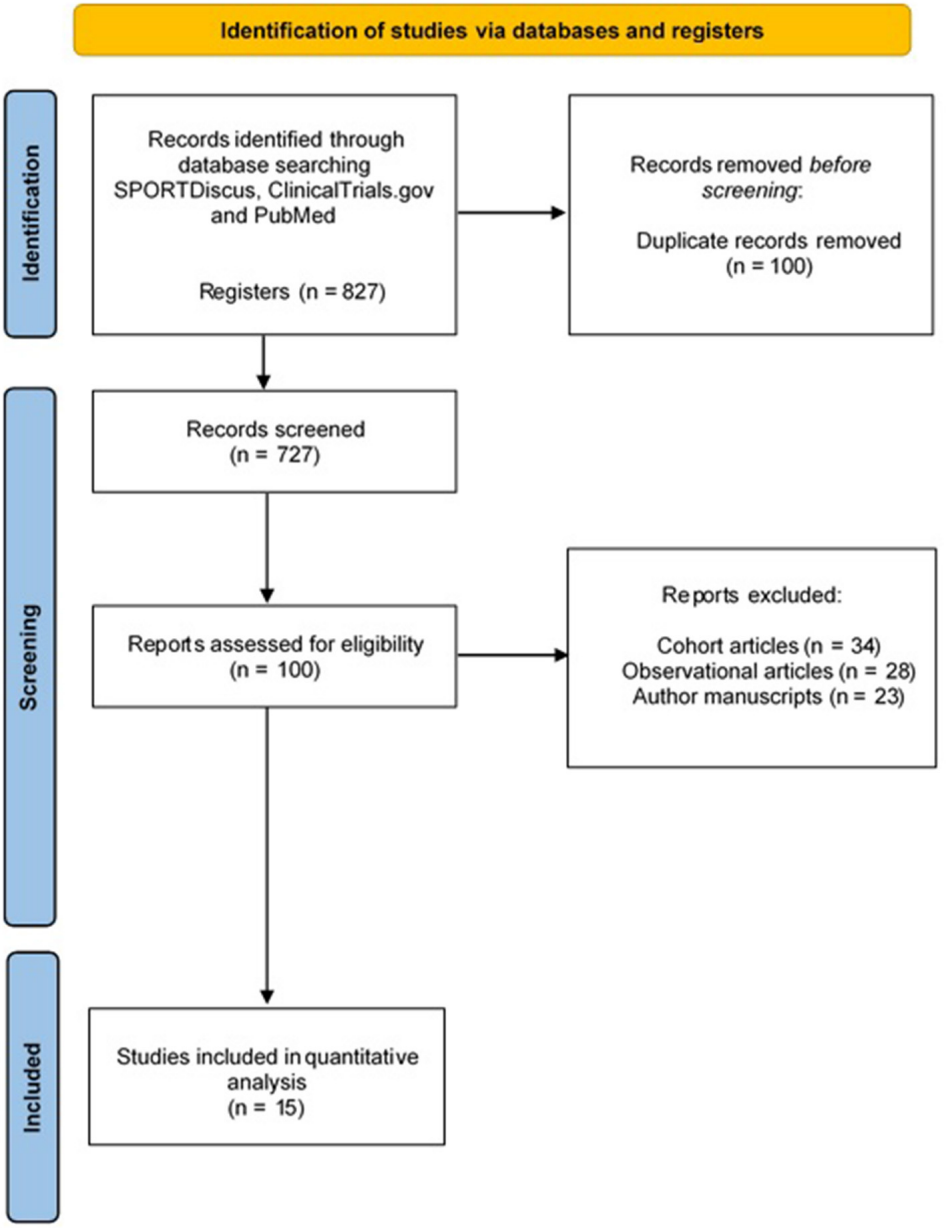
Flow chart of the retrieved and analyzed articles.

The inclusion criteria of the studies were randomized clinical trials whose intervention involved measurable or quantifiable activity or exercise (studies in which only advice to have an active pregnancy was provided were not selected), prenatal depression outcomes (depression symptoms or diagnosed depression) measured, and different characteristics of an exercise program provided. The selection process followed for the reviewed articles is captured in Figure 1 (Page et al., 2021).

Regarding the parameters studied, prenatal maternal depression expressed quantitatively (scale) and/or categorically (maternal depression yes/no) was the primary outcome extracted from the included studies. To determine the extent of the effects of exercise on the health of pregnant women, other pregnancy outcomes were examined as secondary outcomes (sociodemographic and physiological maternal variables and newborn outcomes, among others).

The data extracted from each of the included studies were the author(s) and publication year, country in which the study was conducted, number of participants, details of the type of exercise program, primary and secondary variable(s) analyzed and cointervention, if applicable (Table 1).

### Statistical Analysis

Meta-analysis was performed separately by a different expression of depression variable. First, when the depression variable was expressed as a continuous variable, such as a score obtained by a questionnaire, the overall confidence interval (CI) was calculated using the standardized mean difference (Hedges et al., 2010).

Second, when depression was expressed as a categorical variable (yes/no), the number of events present in each study group and its relative risk (RR) were recorded, and the total sum of the RR was calculated using a random effects model (Higgins and Thompson, 2002).

In both analyses (dichotomous and continuous) the compensated average was established by assigning each study a weight relative to its sample size or number of events that contributed to the entire study (weight) or, in short, the information burden that each of them contributes. To quantify the heterogeneity present in the results, the *I*^2^ statistic was used, which indicates the proportion of variability observed in the effect of the intervention (between studies) that is due to the heterogeneity between studies and is not random. The following thresholds were used for the *I*^2^ statistic: low = 25%, moderate = 50%, and high = 75% (Daley et al., 2018).

One approach previously used to solve the problem of high heterogeneity has been to split the studies into subgroups based on some characteristics that could explain the variability of the studies (Ferreira González et al., 2011). However, in our case, given the limited number of the studies identified for the review, we have opted to present all of the examined studies in each analysis as we understand that this approach provides a more comprehensive view of the study.

### Quality of Evidence Assessment and Risk of Bias

Despite being often mistaken as interchangeable terms, “quality” and “risk of bias” are, in fact, distinct but related concepts that should, thus, be differently addressed (Gunnell et al., 2020). In the present study, the assessment of quality (i.e., the degree to which studies are conducted in alignment with the highest possible standards) was performed by means of the Grading of Recommendations Assessment, Development and Evaluation (GRADE) technique (Guyatt et al., 2008), and studies that were rated as having high or moderate quality were included.

On the other hand, risk of bias (i.e., the degree to which potential biases may have led to underestimation or overestimation of an effect) was assessed following the Cochrane Handbook (Higgins et al., 2020).

## Results

**Figure 2:**
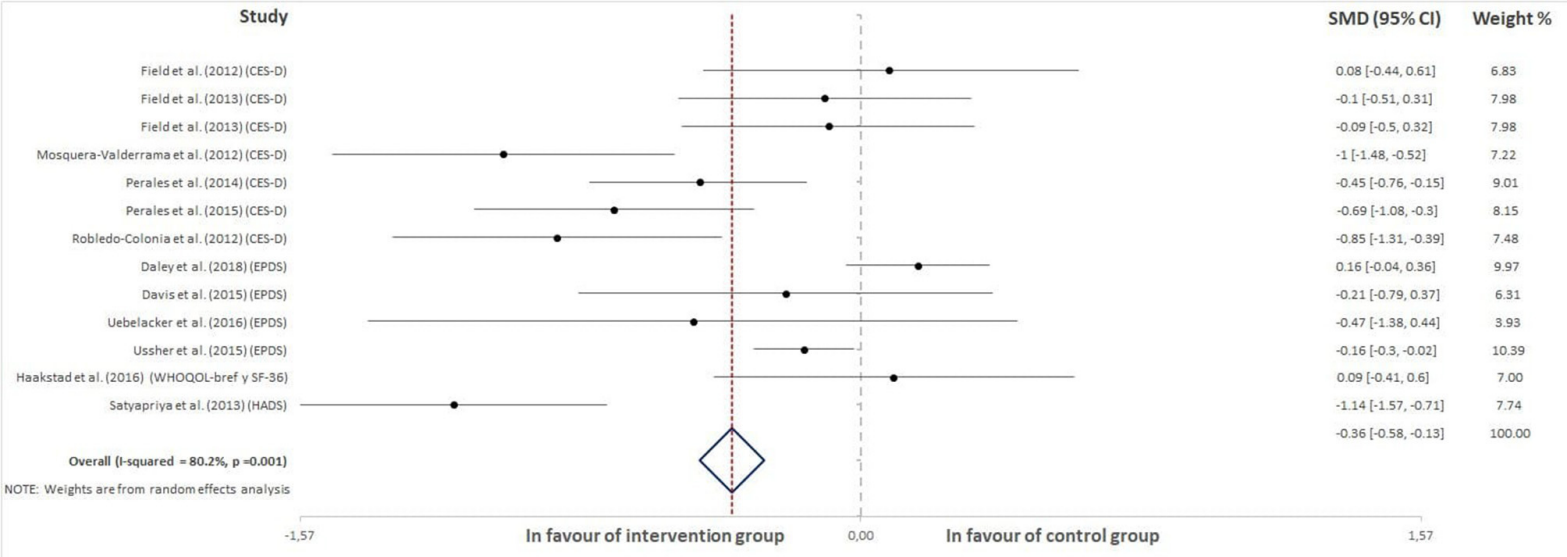
Effect of exercise on the score obtained for the participants on the administered questionnaires.

**Figure 3:**
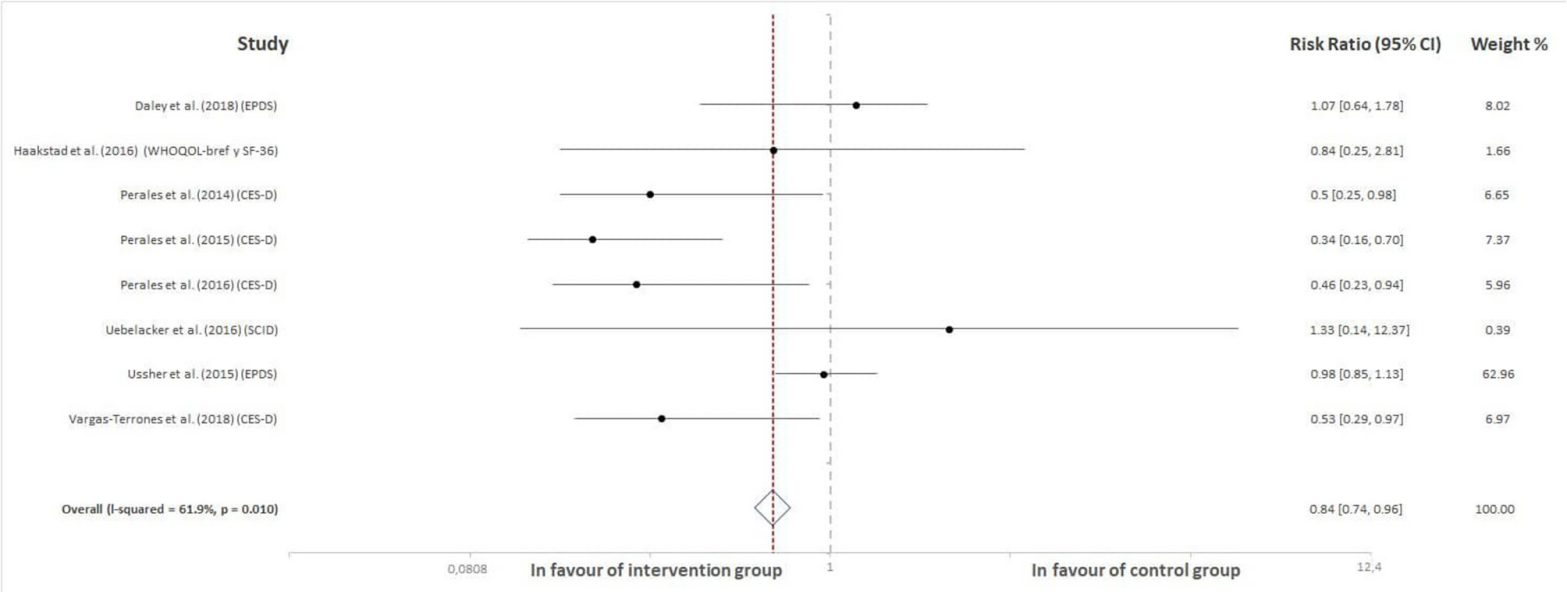
Effect of exercise programs during pregnancy on the probability of experiencing prenatal depression.

From a total of 827 retrieved articles, 727 were excluded for not meeting any of the inclusion criteria (Figure 1). In addition, 85 articles were excluded because they were not completely randomized clinical trials (i.e., they lacked a randomization process). Finally, 15 studies were included for analysis. Thirteen of them reported participants' depression or depression symptoms as a continuous variable (i.e., a score obtained in a questionnaire), and these studies are presented in Figure 2. On the other hand, in eight of them, depression was treated as a categorical variable reporting the number of women showing depression symptoms in each group (control and intervention). These studies are displayed in Figure 3. Last, six studies reported information about depression as both a continuous variable (score obtained in a questionnaire) and a dichotomous variable (suffering/not suffering depression). These works have, thus, been included in both Figures 2, 3 (Perales et al., 2014, 2015; Ussher et al., 2015; Haakstad et al., 2016; Uebelacker et al., 2016; Daley et al., 2018).

Among the 15 articles included in the meta-analysis, 14 included sessions supervised by professionals in the field. The exercise sessions (both supervised and not) were designed for low-to-moderate intensity or moderate-to-high intensity exercise and were not performed more than three times per week. The session durations varied from 20 to a maximum of 75 min for a session.

### Effect of Exercise on the Score Obtained for the Participants on the Administered Questionnaires

Thirteen studies were included in this analysis. The results revealed a negative association between exercise practice during pregnancy and the scores obtained for the questionnaires that were used to measure depression in pregnant women (ES = −0.36, 95% CI = −0.58, −13, *I*^2^ = 80.2%, P_heterogeneity_ = 0.001). Figure 2 shows the forest plot corresponding to the present meta-analysis.

### Effect of Exercise on the Number and Percentage of Depressed Pregnant Women in the Study Groups

A total of eight studies were included in this analysis. The global effect of exercise was evident in the number and percentage of pregnant women when those who were considered depressed in the intervention groups were compared with those in the control groups. Specifically, the total RR compensated was 0.84 (95% CI = 0.74, 0.96, *I*^2^ = 61.9%, P_heterogeneity_ = 0.010). These outcomes indicate that women who remain inactive present a 16% greater probability of experiencing prenatal depression. Figure 3 shows the forest plot corresponding to the present meta-analysis.

### Risk of Bias Assessment

**Figure 4:**
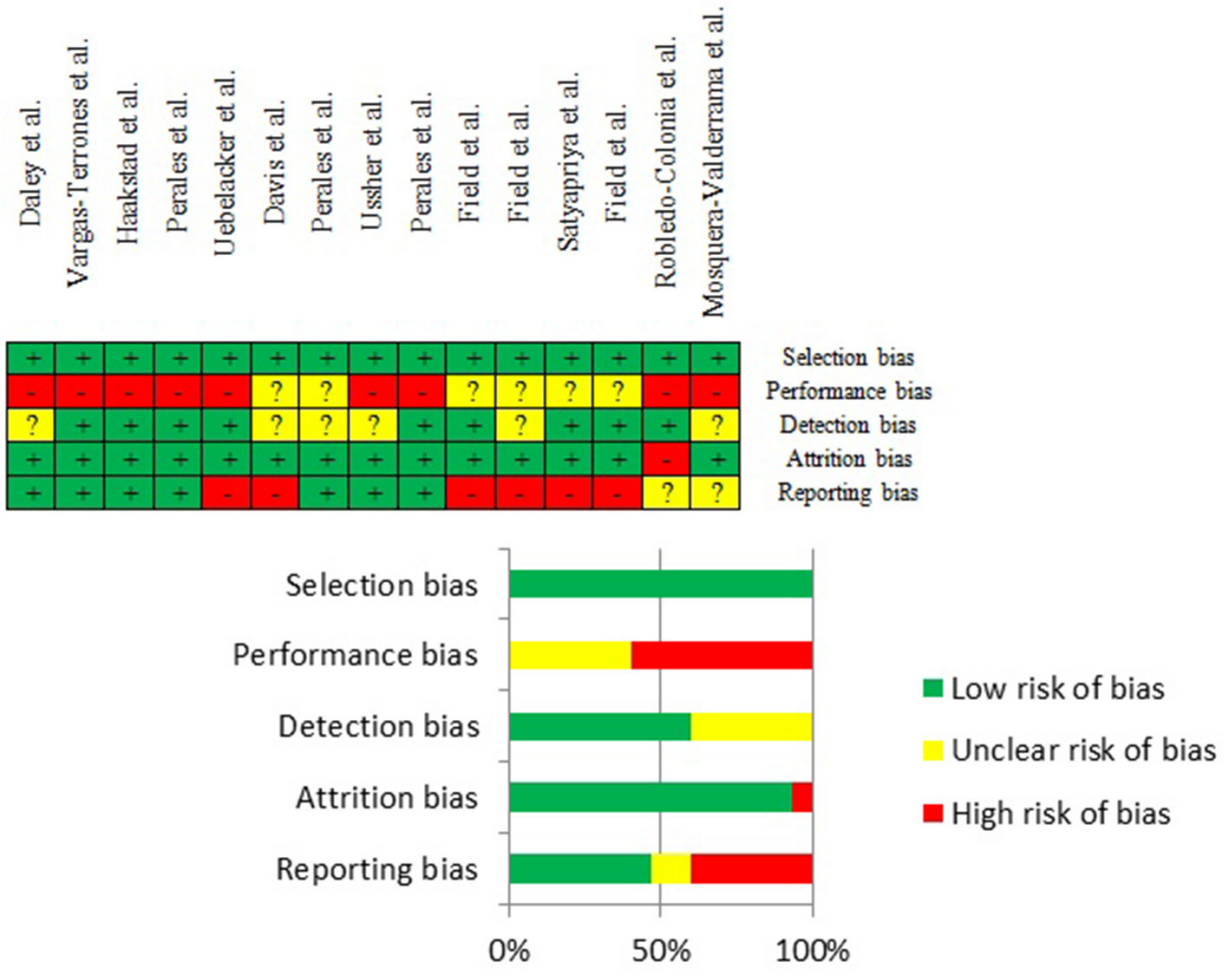
Risk of bias summary.

Overall, the quality of evidence ranged from low to high (see Figure 4; Table 2).

**Table 2:** Risk of bias of reviewed studies.

**Articles/Bias**	**Selection bias**		**Performance bias**		**Detection bias**		**Attrition bias**		**Reporting bias**	
	**Risk**	**Text where it is located**	**Risk**	**Text where it is located**	**Risk**	**Text where it is located**	**Risk**	**Text where it is located**	**Risk**	**Text where it is located**
Daley et al. (2018)	Low	“At the first session, participants were randomly assigned (based on a computer-generated code)…”	High	“It was not feasible to mask participants or researchers to group allocation.”	Unc.	“…higher self-reports of activity in the physical activity group compared with the control group may be biased by knowledge of allocation.”	Low	“When using an intention to treat approach it is acceptable to exclude patients' data, without risking bias…”	Low	The study protocol is available and all of the study's pre-specified (primary and secondary) outcomes that are of interest in the review have been reported.
Vargas-Terrones et al. (2019)	Low	“A simple randomization was performed with the Epidat V.3.1 program to allocate the participants into two groups in order of entry: intervention group (IG) and control group (CG).”	High	The nature of the intervention prevented the study from blinding participants.	Low	“Participants were not involved in the design, recruitment, and conduct of the study.”	Low	“An analysis by intention-to-treat was also performed using two different methods.”	Low	The study protocol is available and all of the study's pre-specified (primary and secondary) outcomes that are of interest in the review have been reported.
Haakstad et al. (2016)	Low	“Allocations were sealed in opaque numbered envelopes following a simple computer-based randomization program.”	High	The nature of the intervention prevented the study from blinding participants.	Low	“In order to treat the two groups identically apart from for the experimental intervention, the controls underwent all tests and completed the same interview as the exercise group, also with respect to assessment of total physical activity level and exercise habits.”	Low	“The principal analysis was done on an intention to treat basis…”	Low	The study protocol is available and all of the study's pre-specified (primary and secondary) outcomes that are of interest in the review have been reported.
Perales et al. (2016)	Low	“Thereafter, they were randomly assigned to a standard care (control, initial *n* = 121) or intervention group (exercise, *n* = 120) using a computer-generated list of random Numbers.”	High	“The study participants and the qualified fitness instructors who supervised the exercise sessions were not blinded to the group allocation.”	Low	“The researchers responsible for assessing eligibility, baseline measures, or outcome assessment were blinded to the group allocation.	Low	“All the analyses were performed using the Statistical Package for Social Sciences program version 22.0, and were adhered to the intention-to-treat principle…”	Low	The study protocol is available and all of the study's pre-specified (primary and secondary) outcomes that are of interest in the review have been reported.
Uebelacker et al. (2016)	Low	“Once we received clearance, we re-contacted the participant and randomized her to the prenatal yoga program (PYP) or a perinatal health education control condition.”	High	“Because this is a study of behavioral interventions, participants could not be blind to which intervention they received.”	Low	“Study groups did not differ on any variables.”	Low	No missing data.	High	Cannot locate protocol.
Davis et al. (2015)	Low	“Participants in both conditions completed a clinical interview and baseline self-report questionnaires prior to randomization.”	Unc.	“Interrater reliability for the Yoga Adherence Scale was 98% for the four classes that both research assistants evaluated.”	Unc.	There is no evidence about blinding of outcomes, but everything suggests that it is done.	Low	No missing data.	High	Cannot locate protocol.
Perales et al. (2015)	Low	“A computer-generated list of random numbers was used to allocate the participants into the groups.”	Unc.	“The randomization blinded process (sequence generation, allocation concealment, and implementation) was performed by three different authors.”	Unc.	There is no evidence about blinding of outcomes, but everything suggests that it is done.	Low	No missing data.	Low	The study protocol is available and all of the study's pre-specified (primary and secondary) outcomes that are of interest in the review have been reported.
Ussher et al. (2015)	Low	“An independent statistician generated a randomization list using Stata, with random permuted blocks of random size stratified by recruitment cente, in a 1:1 ratio.”	High	“It was not feasible to mask participants or researchers to group allocation.”	Unc.	There is no evidence about blinding of outcomes, but everything suggests that it is done.	Low	“Analysis was on an intention to treat basis…”	Low	The study protocol is available and all of the study's pre-specified (primary and secondary) outcomes that are of interest in the review have been reported.
Perales et al. (2014)	Low	“For allocation of the participants, a computer-generated list of random numbers was used.”	High	“…due type of intervention blinding of participants was not possible.”	Low	“Randomization process (sequence generation, allocation concealment, and implementation) was made for three different authors in order to facilitate blinding of process and outcomes assessment.”	Low	No missing data.	Low	The study protocol is available and all of the study's pre-specified (primary and secondary) outcomes that are of interest in the review have been reported.
Field et al. (2013a)	Low	“…the depressed pregnant women were randomly assigned to a yoga or a social support group based on a random numbers table.”	Unc.	“…by trained researchers who were blinded to the group assignment and the study hypotheses.	Low	“The groups did not differ on demographic variables and baseline measures.	Low	No missing data.	High	Cannot locate protocol.
Field et al. (2013b)	Low	“The participants were clinically depressed pregnant women who were randomly assigned to either a tai chi/yoga treatment or a control group.”	Unc.	The nature of the intervention prevented the study from blinding participants.	Unc.	There is no evidence about blinding of outcomes, but everything suggests that it is done.	Low	No missing data.	High	Cannot locate protocol.
Satyapriya et al. (2013)	Low	“…the subjects were allocated to two groups (yoga and control) using a computer generated random number…”	Unc.	As this was an interventional study, the participants or the trainer could not be blinded. Attempts were made to mask wherever feasible to reduce the bias.	Low	“The team who did the assessments was not involved in administering the intervention. The statistician who did the randomization and analysis was blind to the source of the data.”	Low	No missing data.	High	Cannot locate protocol.
Field et al. (2012)	Low	“The women were then randomly assigned to a yoga, massage therapy or standard prenatal care control group.”	Unc.	“All assessments were conducted by the trained research associates who were blind to the study' hypotheses and to the group assignment.”	Low	“In addition, the yoga and massage therapy groups did not differ on neonatal outcomes including gestational age and birthweight.”	High	There are missing data (more than 10% of sample).	High	Cannot locate protocol.
Robledo-Colonia et al. (2012)	Low	“Randomization was performed using a permuted block design with a block si e of 10 and exp:con ratios of 5:5, 6:4 or 4:6.”	High	“Participants and therapists administering the intervention were not blinded.”	Low	“The investigators responsible for outcome assessment were blinded to group allocation.”	Low	“Analysis was according to the principle of intention-to-treat.”	Unc.	The variables don't coincide in the protocol and in the article methodology.
Mosquera-Valderrama et al. (2012)	Low	“Después de la realización de estas pruebas, las pacientes fueron asignadas aleatoriamente.”	High	“La principal limitación es que los terapeutas de campo y las participantes no pueden ser cegados a las intervenciones con el entrenamiento físico aeróbico.”	Unc.	There is no evidence about blinding of outcomes, but everything suggests that it is done.	Low	“…destacamos la validez de los hallazgos debido al diseño del estudio, que incorpora varias características que minimizan la posibilidad de sesgo en los resultados, tales como la aleatorización, y el análisis de intención de tratamiento.”	Unc.	The variables don't coincide in the protocol and in the article methodology.

*Unc., unclear*.

Most of the studies exhibited low selection and attrition bias. There was a reasonable number of studies whose risk of detection bias was found to be unclear because studies reported whether outcome assessors had not been blinded to participants. The most common sources of risk of bias were (a) the low likelihood that either study participants or personnel were blinded to the experimental condition (performance bias) and (b) the fact that findings of some outcomes appearing in the protocol of the studies were not published in the analyzed studies (reporting bias).

Despite the risk of bias findings, it was decided to not disregard any study in our analyses. First, with regard to performance bias, because it is acknowledged in the Cochrane Collaboration's Tool (Higgins et al., 2020), blinding is not possible in certain situations (it is usually impossible to blind people to whether a PA program has been followed). Second, relating to the reporting bias, based on data extracted from the included studies, it is unlikely that the remaining outcomes of interest (biological rather than social data) were significantly associated with depression.

## Discussion

The present study aimed to determine the effects of exercise during pregnancy on maternal prenatal depression. The results show a trend toward a small reduction in both the scores of the instruments (questionnaires) that measured the prevalence of depressive symptoms in pregnant women and the number and percentage of women with diagnosed prenatal depression in the intervention groups (physical exercise). It is important to clarify that, during pregnancy, a woman may present depressive symptoms and increased emotional lability but not be diagnosed with prenatal depression although obviously both conditions are closely associated.

Because all the studies investigated healthy pregnant women, the results obtained in these studies are related to depressive symptoms rather than to diagnosed depression. Therefore, from a global perspective, the findings of the present study could be used and have an important clinical application to prescribe groups and supervised exercise during pregnancy as a preventive factor against emotional lability and associated complications. The results of the present study allow us to conclude that supervised exercise during pregnancy constitutes a powerful tool for preventing and reducing prenatal depression.

Regarding the impact of exercise during pregnancy, the results suggest that there is a positive association between an active pregnancy and a more balanced and adequate emotional state. Because depressive symptoms begin early in pregnancy (first trimester), the supervised exercise program designed for healthy pregnant women should also begin early, once it is known that there are no obstetric contraindications to exercise during pregnancy.

The study designed by Perales et al. (2014) deserves particular attention; the article included 167 pregnant women and noted that, although depression levels were similar in both groups (slightly higher in the intervention group) at the beginning of pregnancy, by the end of pregnancy there was a significant difference (reduction) in favor of the intervention group (exercise) compared with the control group in relation to both the questionnaire values (CES-D questionnaire) and the number and percentage of pregnant women considered depressed. Overall, this result confirms the positive impact of a supervised exercise program during pregnancy on the emotional state of pregnant women.

It is interesting to analyze what factors present in exercise programs can be associated with the emotional response of pregnant women and determine an improvement in emotional state. In this sense, we find that both the supervision of exercise by a professional and the adherence of the participants to the program were relevant factors to increase the positive impact of exercise on improving emotional state. This positive association between exercise and the prevention of maternal prenatal depression has been demonstrated by some studies with supervised exercise programs and high adherence of the participants (Field et al., 2012; Perales et al., 2014, 2015, 2016; Ussher et al., 2015; Haakstad et al., 2016; Uebelacker et al., 2016; Vargas-Terrones et al., 2019).

The other important aspects seem to include the type of exercise and the activities implemented in the sessions. Thus, some studies include a combined practice with mixed activities (aerobic resistance, muscle strengthening, coordination, pelvic floor, flexibility) and show good results (Mosquera-Valderrama et al., 2012; Robledo-Colonia et al., 2012; Perales et al., 2014, 2015, 2016; Haakstad et al., 2016; Vargas-Terrones et al., 2019). Additionally, there was a reduction in prenatal depression in studies that used yoga (Davis et al., 2015) or tai-chi (Field et al., 2013a) as an intervention program; however, it is important to highlight that there is no absolute consensus as to which type of exercise reduces these symptoms the most.

In a recent RS-MA, Davenport et al. (2018) found a positive influence of supervised exercise on the reduction of depressive symptoms during pregnancy. Similarly, there are other recent systematic reviews that conclude that supervised exercise with low-to-moderate intensity is a preventive measure against postpartum depression as well as gestational depression (Shivakumar et al., 2011; Daley et al., 2015; Gong et al., 2015).

As it is been shown that women who were inactive during pregnancy were 16% more likely to suffer prenatal depression, future studies will focus on comparing the effect of antidepressants during pregnancy with exercise during the same period. However, more studies examining the effect of exercise during pregnancy are needed to contribute to understanding our research question.

The limitations of this study were the scarcity of scientific literature about exercise and prenatal depression because there are more papers that study postnatal depression than prenatal depression, the lack of consensus on the depression scales used (there were four different scales with four different measured scores), and the variability of the exercise programs performed in all studies. The results of the present study demonstrate that aerobic and supervised exercise during pregnancy could be a tool to prevent depressive symptoms as well as diagnosed prenatal depression.

## Data Availability Statement

The original contributions presented in the study are included in the article/supplementary material, further inquiries can be directed to the corresponding author.

## Author Contributions

MS-P was responsible for the design of the review study, contributed to the data selection and analysis, and the preparation of the manuscript. EF led data selection and analysis and contributed to manuscript writing. CS-J and JG-A contributed to the data selection and analysis. JP-T contributed to the data selection and analysis and the preparation of the manuscript. RB contributed to the design of the study and the manuscript writing. IR was mainly responsible for the design of the review study and the data analysis and led the manuscript writing. All authors contributed to the article and approved the submitted version.

## Conflict of Interest

The authors declare that the research was conducted in the absence of any commercial or financial relationships that could be construed as a potential conflict of interest.
